# Isolation and cDNA characteristics of *MHC-DRA* genes from gayal (*Bos frontalis*) and gaytle (*Bos frontalis* × *Bos taurus*)

**DOI:** 10.1080/13102818.2014.986128

**Published:** 2014-12-16

**Authors:** Yongke Sun, Xiaomin Zhang, Dongmei Xi, Guozhi Li, Liping Wang, Huanli Zheng, Min Du, Zhaobing Gu, Yulin Yang, Yuai Yang

**Affiliations:** ^a^ Faculty of Animal Science and Technology, Yunnan Agricultural University, Kunming, Yunnan, P.R. China

**Keywords:** gayal (*Bos frontalis*), gaytle (*Bos frontalis* × *Bos taurus*), *DRA* gene, functional domains

## Abstract

The mammalian major histocompatibility complex (MHC) plays important roles in pathogen recognition and disease resistance. In the present study, the coding sequence and the 5′- and 3′-untranslated regions of MHC class II DR alpha chain (the DRA gene) from rare gayal and gaytle were cloned and analyzed to dissect structural and functional variations. The nucleotide and amino acid sequences for the *DRA* genes in gayal (*Bofr-DRA*) and gaytle (*Bofr* × *BoLA-DRA*) were almost identical to those for cattle and yak (99%). Compared to yak, two amino acids substitutions in the signal peptide (SP) domain for gayal were found within all *Bos* animals. Except for only one replacement in the amino acid within the α2 domain of the DRA protein in gayal, the additional residues were highly conserved across the species investigated. The 20 peptide-binding sites (PBS) of *Bofr-DRA* and *Bofr* × *BoLA-DRA* were essentially reserved in the α1 domain among all species investigated. The lesser degree of substitution in *Bofr-DRA* is concordant with the concept that the *DRA* gene is highly conserved among all mammals. The very high degree of conservativity of the *DRA* gene among ruminants, including gayal, suggests its recent evolutionary separation.

## Introduction

The immune system is a function of biological structures and processes within an organism that protects against diseases by recognizing and responding to pathogens and antigens.[1] The mammalian major histocompatibility complex (MHC) is a group of closely linked and highly variable genes and gene clusters.[2] The cattle MHC, which is called bovine leukocyte antigen (BoLA), plays a key role in the defense against infectious diseases and regulates antigen presentation by encoding proteins that take part in the innate and adaptive immune responses to intruding pathogens.[3,4] The MHC genes are associated with susceptibility or resistance to many diseases for cattle and sheep.[5,6] Thus, the MHC genes could be indicated as primary candidates for the investigation of genetic variation for the purpose of marker-assisted breeding.

The gayal or mithun (*Bos frontalis*) is a rare semi-wild bovine species naturally inhabiting Indo-China.[7,8] It has a chromosome complement of 2*n* = 58,[9–11] which differs from those of their counterparts such as cattle (*B. taurus*, 2*n* = 60) and gaur (*B. gaurus*, 2*n* = 56).[12] Gayal often consumes tree and bamboo leaves, grasses, reeds and other local plants and shows a very wide range of adaptations under the harsh conditions which range from cold to tropical regions.[13,14] Moreover, the quality of meat, including tender and muscle fibre diameter, from gayal is better than in local cattle.[15,16] Therefore, the hybrid between gayal (♂) and cattle (♀), which is also called gaytle (gayal × cattle), presents better meat traits than cattle and can meet the diversity demand for consumers. Due to the remoteness of their habitats, as well as socio-political and ecological factors, gayal is one of the least studied ruminants.[17–20]

Until now, the *MHC-DRA* gene has been cloned and sequenced in European cattle [21] and zebu,[22] yak and yakow,[23,24] buffaloes,[25] goats,[26] sheep,[22,27] donkeys,[28] pigs,[29] mice,[30] cats [31] and macaques.[32] So far, the *MHC-DRA* from gayal and gaytle has not been deposited in the GenBank database (http://www.ncbi.nlm.nih.gov/). These native animals are more common sources of meat and milk than cattle in Indo-China. They are considered to be less susceptible than cattle to some endemic diseases.[33,34] In the present study, we have isolated and characterized full-length cDNAs from gayal and gaytle and compared those with the sequences of *MHC-DRA* from different animal species in order to inspect the genetic factors for disease susceptibility and resistance.

## Materials and methods

### Samples collection, RNA extraction and first-strand cDNA synthesis

Liver RNA samples from three mature gayals (*B. frontalis*) and three mature gaytles (*B. frontalis* × *B. taurus*) were collected at the slaughter house, Gongshan County (Yunnan Province, China). The samples were snap frozen and kept in liquid nitrogen during storage and transportation, and then stored at −80 °C. The total RNA was isolated using a Total RNA Extraction Kit (Beijing Tiangen Biotech Co., Ltd., China). DNase I treatment of the total RNA was performed before the cDNA was constructed using RevertAid™ First Strand cDNA Synthesis Kits (Fermentas Inc., USA) following the manufacturer's instructions.

### Primers and PCR amplification of the DRA gene

The *MHC-DRA* genes from gayal and gaytle were isolated using forward primer (5′-CGAGACACCGAAGAAGAAAAT-3′) and reverse primer (5′-GGAGGGAAAACCAATACAAGAA-3′), which were also successfully used to amplify *DRA* sequences from buffalo, yak and yakow.[20,21] Polymerase chain reaction (PCR) was conducted using Bioer Life Express Thermocycler (Bioer Technology Co., Ltd., China) using 2 μL of cDNA template, 12.5 μL of 2× PCR Power Mix (Beijing Zoman Biotechnology Co., Ltd., China), 2 μL of 10 pmol/μL of each primer and 8.5 μL of double distilled water in a total reaction volume of 25 μL. The PCR conditions started with an initial denaturation temperature of 94 °C for 4 min, followed by 36 cycles of the following steps: 94 °C for 1 min, 56 °C for 1 min and 72 °C for 1 min, with a final extension of 10 min at 72 °C. Visualization of amplified products was performed on agarose gel stained with ethidium bromide. The PCR products were sequenced bi-directionally using an ABI373X DNA analyzer (Applied Biosystems Inc.) at the Sango Biotechnology Company (Shanghai, China).

### Sequences analysis

The cDNA sequence prediction was conducted using the GenScan software (http://genes.mit.edu/GENSCAN.html).[35] The theoretical isoelectric point (pI) and molecular weight (Mw) of the putative DRA proteins were computed using the Compute pI/Mw Tool (http://www.expasy.org/tools/pi_tool.html).[36] The DNAStar v5.2.2 program and MEGA v5.0 software package with the neighbour-joining method [37] were used to conduct sequences alignment and to construct a phylogenetic tree.

## Results and discussion

### Identification and characterization of the *Bofr-DRA* and *Bofr × BoLA-DRA*

**Figure 1.**
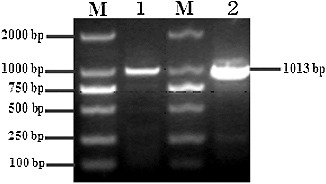
RT-PCR result for the *DRA* genes from gayal (1) and gaytle (2). M: DL2000 DNA marker.

The resulting PCR products of 1013 bp for both *Bofr-DRA* (gayal: *B. frontalis*) and *Bofr* × *BoLA-DRA* (gaytle: *B. frontalis* × *B. taurus*) genes were obtained through reverse transcription PCR (RT-PCR) using liver cDNA as a template (Figure 1). The fragment of 972 bp including the complete coding region (762 bp) and parts of the 5′- and 3′-untranslated regions (27 and 183 bp, respectively) of the *MHC-DRA* gene was effectively sequenced and deposited into the GenBank database with accession numbers KF981723 and KF981724, respectively. Compared with the bovine *MHC-DRA* (GenBank accession number D37956), the complete coding region of *Bofr-DRA* and *Bofr* × *BoLA-DRA* genes (762 bp) encoded a polypeptide of 253 amino acids with a predicted Mw of 28,425 and 28,407 Da (pI 5.41), respectively.

### Sequence alignment and comparison

Table 1 presents the nucleotide sequences and amino acid alignments. The *Bofr-DRA* and *Bofr* × *BoLA-DRA* genes had the closest similarity (99%) to the *DRA* gene of cattle, zebu, yakow and yak, with high level of similarity with the respective gene in buffalo, goat, sheep, pig, dog, macaque, human, cat and mouse. In addition to the signal peptide (SP), other functional domains, including the α1, α2, connecting peptide (CP), transmembrane (TM) and cytoplasmatic (CY) regions, are highly conserved, particularly the sites associated with biological function in these *DRA* genes.[25] Importantly, the SP domain has been reported to have the most variable region of the molecule across the species.[25] However, there was high similarity among *Bofr-DRA*, *Bofr* × *BoLA-DRA* genes and those of other ruminants.

**Table 1.** Sequence similarity comparison at the nucleotides and amino acids (within parentheses) sections for the signal peptide (SP), α1, α2 and connecting peptide/transmembrane/cytoplasmatic (CP/TM/CY) domains of the *DRA* genes from different species compared to those for gayal (*B. frontalis*, up) and gaytle (*B. frontalis* × *B. taurus*, down).

	Domains				
Species	SP	α1	α2	CP/TM/CY	Full cDNA
Gaytle	100.0 (100.0)	100.0 (98.0)	99.0 (98.0)	100.0 (100.0)	99.0 (99.0)
	NC	NC	NC	NC	NC
					
Cattle	100.0 (100.0)	99.0 (98.0)	100.0 (98.0)	100.0 (100.0)	99.0 (99.0)
			99.0 (100.0)		99.0 (100.0)
					
Zebu	100.0 (100.0)	99.0 (98.0)	100.0 (98.0)	100.0 (100.0)	99.0 (99.0)
			99.0 (100.0)		99.0 (100.0)
					
Yakow	100.0 (100.0)	99.0 (98.0)	99.0 (100.0)	100.0 (100.0)	99.0 (100.0)
			100.0 (98.0)		99.0 (99.0)
					
Yak	98.0 (91.0)	99.0 (98.0)	99.0 (100.0)	100.0 (100.0)	99.0 (99.0)
			100.0 (98.0)		99.0 (98.0)
					
Buffalo	95.0 (95.0)	98.0 (96.0)	99.0 (98.0)	96.0 (94.0)	98.0 (97.0)
			98.0 (100.0)		97.0 (97.0)
					
Sheep	98.0 (95.0)	92.0 (85.0)	97.0 (94.0)	98.0 (98.0)	96.0 (92.0)
			97.0 (95.0)		96.0 (93.0)
					
Goat	97.0 (91.0)	95.0 (92.0)	97.0 (95.0)	98.0 (100.0)	96.0 (95.0)
			96.0 (96.0)		96.0 (96.0)
					
Pig	70.0 (60.0)	86.0 (84.0)	86.0 (81.0)	88.0 (80.0)	86.0 (80.0)
			86.0 (82.0)		86.0 (81.0)
					
Macaque	75.0 (54.0)	87.0 (83.0)	84.0 (82.0)	83.0 (80.0)	85.0 (81.0)
			84.0 (84.0)	83.0 (78.0)	85.0 (81.0)
					
Human	76.0 (58.0)	86.0 (82.0)	85.0 (82.0)	76.0 (75.0)	85.0 (80.0)
	81.0 (62.0)	86.0 (83.0)	85.0 (84.0)	83.0 (78.0)	85.0 (81.0)
					
Dog	71.0 (60.0)	90.0 (88.0)	86.0 (85.0)	85.0 (76.0)	86.0 (78.0)
	81.0 (62.0)	86.0 (83.0)	87.0 (84.0)	87.0 (78.0)	86.0 (81.0)
					
Cat	72.0 (26.0)	87.0 (85.0)	87.0 (81.0)	88.0 (80.0)	86.0 (77.0)
	72.0 (25.0)	90.0 (87.0)	86.0 (85.0)	85.0 (76.0)	86.0 (78.0)
					
Mouse	61.0 (33.0)	76.0 (73.0)	78.0 (74.0)	76.0 (60.0)	77.0 (67.0)
			78.0 (75.0)		77.0 (68.0)

Note: Identical data of similarity comparison of gayal or gaytle with one species were omitted and shown only in one line. NC: not compared.

**Figure 2.**
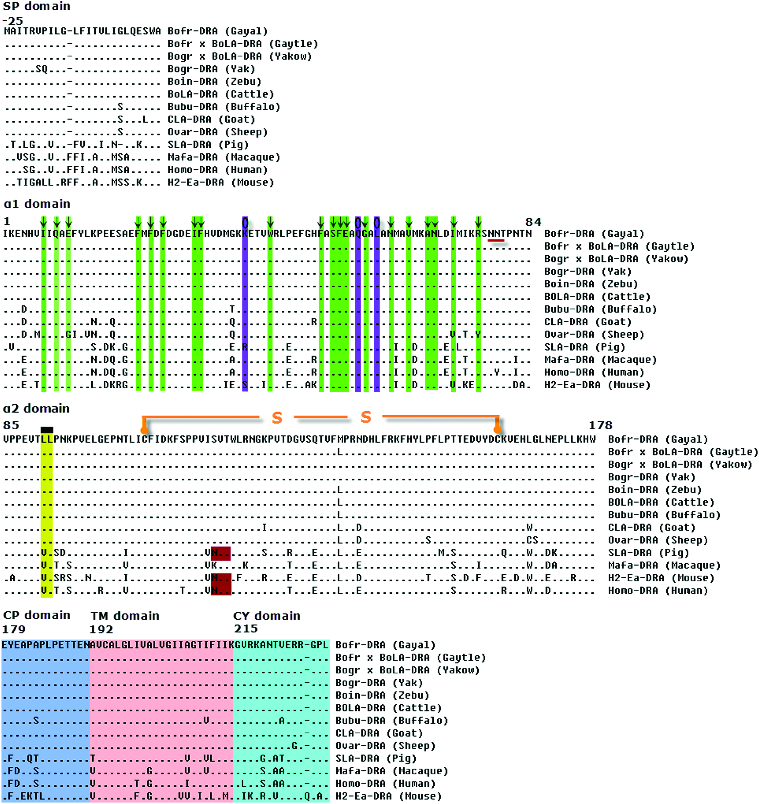
Alignment of the protein encoded by the *DRA* genes in gayal (*Bos frontalis*), gaytle (*B. frontalis* × *B. taurus*), European cattle (*B. taurus*, D37956), zebu cattle (*Bos indicus*, FM986338), yak (*Bos grunniens*, JQ911700), Chinese yakow (*B. grunniens* × *B. taurus*, JQ347519), buffaloes (*Bubalus bubalis*, DQ016629), goats (*Capra hircus*, AB008754), sheep (*Ovis aries*, M73983), macaques (*Macaca mulatta*, EF208826), humans (*Homo sapiens*, NM_019111), pigs (*Sus scrofa*, M93028) and mice (*Mus musculus*, NM_010381). A hyphen (-) indicates amino acid identity and an asterisk (*) indicates a gap inserted for maximum alignment. Arrows (↓) within the green column indicate the amino acid positions constituting part of peptide-binding site (PBS). A circle (О) with a blue column indicates a conserved site for T-cell receptor interaction and putative N-linked glycosylation sites are underlined. A rectangle sign (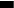) denotes the position of residues associated with binding of CD4 molecules. S-S indicates a disulphide bond between two cysteine residues. Proposed DRA locus specific motifs within connecting peptide (CP), transmembrane (TM) and cytoplasmatic (CY) domains are indicated by pink, green and yellow areas, respectively.

The entire SP, α1, α2 and CP/TM/CY domains from DRA molecules from gayal and gaytle were almost identical to those of cattle, except the mutation in position 138 in the amino acid chain within the α2 domain (P. M138L, Figure 2). However, compared with yak, there was minimal mutation of the *Bofr-DRA* and *Bofr* × *BoLA-DRA* genes, with only four single nucleotide substitutions within the coding regions, resulting in two amino acid replacements in the sixth and seventh position within the SP domain (p. V6S and p. P7Q).

The 20 amino acids which are located in positions 7, 9, 11, 22, 24, 26, 31, 32, 43, 51, 53, 54, 55, 58, 62, 65, 68, 69, 72 and 76 of the peptide-binding site (PBS), as reported by Soen et al.,[38] were highly conserved within the α1 domain (Figure 2). Compared to other MHC class II gene families such as the *DRB*, *DQA* and *DQB*, which are highly variable at the PBS,[39–42] the *DRA* gene appears to be highly conserved for residues forming the PBS region in different mammals, including primates. It seems that selection pressure has led to conservation for these specific functional regions in the *DRA* gene. However, there was evidence of variability in the first domain exon (α1) of *DRA* of ruminants.[22,43] These results could emphasize the complexity and function of the *DRA* gene, particularly in the PBS region. The cysteine residues that build an interchain disulphide bond between positions 107 and 163 were also stable and conserved. In gayal, gaytle and other animals including cattle, zebu, yak, yakow, buffaloes, sheep, goats and macaques, the DRA molecules have only one potential N-linked glycosylation site (78–80 amino acids) in the α1 domain as opposed to two that occur in pigs, humans and mice, with the second site located in the α2 domain (118–120 amino acids marked by the reddish box in Figure 2).[21,26] The deprivation of one glycosylation site in the α2 domain for gayal and other ruminants such as cattle and yak is due to the replacement of an asparagine residue with serine. It is not known whether the glycosylation site in the α2 domain may have an important role in the immune response and the response against specific antigens, although its lack presents a specific molecular characteristic of ruminant *DRA* genes. There were no substitutions in other important functional sites such as T lymphocytes receptor recognition site (positions 39, 57 and 60) and CD4 molecule-binding site (positions 91–92) in gayal, gaytle, yak, Chinese yakow, zebu, cattle, buffaloes, sheep and goats.

### Phylogenetic analysis based on neighbour-joining method

**Figure 3.**
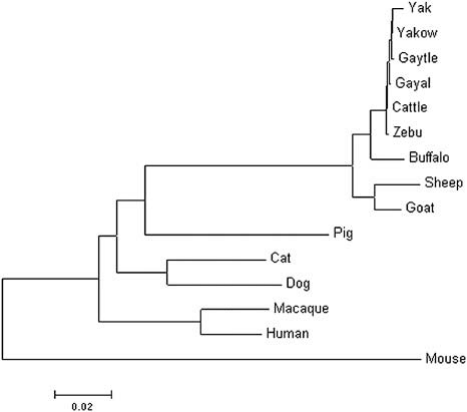
Phylogenetic tree for the *DRA* genes from gayal, gaytle, European cattle, zebu, yak, Chinese yakow, buffaloes, goats, sheep, macaques, humans, pigs, cats, dogs and mice.

From the phylogenetic tree analysis (Figure 3), it appears that the *DRA* genes of ruminant and non-ruminant species evolved independently, since the species they originate from have split into different branches over the evolutionary time. It is also apparent that yak and cattle are most closely related species to gayal.

In the present study, the loss of mutation in *Bofr-DRA* and *Bofr* × *BoLA-DRA* is concordant with the *DRA* gene being conserved within ruminants and all mammalian species. As a result, this could be used for the identification of shared antigenic sites of similar pathogens or the recognition of very specific antigens which may be common to most of the species. Moreover, the close similarity of the *DRA* gene among ruminants, including gayal, may be ascribed to recent separation in evolutionary process and/or similar selection pressure which the ruminants have suffered during evolution.

## Conclusions

The *DRA* genes of gayal and gaytle have been isolated and characterized for the first time, thereby enlarging cognizance of the *MHC-DRA* in rare ruminants. It points out that the *Bofr-DRA* gene is highly conserved, particularly in the first domain exon as in most mammals. In the future, it would be more interesting to dissect *Bofr-DRB*, which probably contains most of the polymorphism within the MHC-DR molecule.
